# The Problem of Influenza

**Published:** 1919-03-15

**Authors:** 


### THE PROBLEM OF INFLUENZA.

Few discussions interest the public more than those which concern its health, and none more than those relating to the fluctuations and prevention of such severe and far-reaching epidemics as lately characterised this so-called influenza. Never in the history of disease have people, in so short a time, been subjected to so much gratuitous advice, heard vaguely of so many discoveries, and suffered so many apparent disappointments. The blame cannot be laid upon research workers either in the war zones or at home, but upon Press activity in exploiting a popular anxiety and attempting to produce or even forestall epoch-making announcements upon material apart from facts. Take what disease we will there is no finality; each piece of research provides experience and, according to the measure of its success, opens the way to fresh advances. The complications, elaborate technique, and great scope of modern investigations in no way lend themselves to the rapid production of landmarks such as the public have been led to expect. It is the duty of the profession to act in accordance with the latest practical information, but it is both unwise and unnecessary that the layman should be repeatedly supplied with fragments from the advancing edge of medical science from which he can assume a great deal but understand little. Judging from the papers of the last few months there must, to the lay mind, appear to be one paramount urgency. The causal germ of influenza must be defined, isolated, and cultivated before any efficient preventive measures can be available.

Let us briefly review the actual situation. The first announcement tells of a filter-passing virus, carried by the blood stream of infected people, and an exact parallel to that discovered in trench fever. The original observers suggest that in it has been found an ultra-microscopic organism, and propose to attempt complete isolation of these minute germs. The second investigation really consists of repeated cultivation from the sputum and fauces of bacilli, known ever since 1892 as the "influenzal" type. The commonest is that named after Pfeiffer, and these organisms also produce soluble filter-passing poisons capable of producing some at least of the milder symptoms of disease. From both sources either a virus or a toxin appears in the blood, and our object is to prepare an absolutely efficient anti-serum, as in the case of diphtheria. Such accomplishment will be the real discovery, a discovery of method, but at present it is found impossible sufficiently to immunise animals against the toxins obtained by either set of workers; in fact, it is doubtful whether animals suffer from the human influenza at all. Vaccine treatment with Pfeiffer's bacillus has, however, both in Army and civilian life, met with much greater prophylactic success than appears to be generally known, and this fact, when compared with the doubtful and conflicting evidence on the ultra-microscopic germs, makes a very strong case for the better-known organism and a simpler explanation.

If Pfeiffer's bacillus be the primary cause, there is much to be thankful for. It is well known as one of the common catarrh-producing organisms, of low vitality outside the body, but showing occasional periods of increased virulence corresponding to the milder pre-war epidemics. During the last few years humanity has repeatedly faced those conditions of strain or crowding which produce that weak bodily resistance and the chance of repeated transference favourable to the attacks of any organism. Weather, lack of heating, poor quality of food, and many other circumstances have played their part. The immediate cause of pneumonia and other complications has been repeatedly identified with the usual strepto- and pneumococci of the respiratory tract, potentially pathogenic germs which may spring into activity when the bodily strength is lowered by any means whatever. The recent epidemics would appear to be but exaggerations of formerly known habits of the bacilli, fostered by the conditions of war, and there is every reason to suppose that the well-known influenzal group is at the root of much of the recent sickness. Some authorities claim to find and cultivate the germ in 70 to 80 per cent. of cases, and we must remember it is not at all easy to isolate this germ in the laboratory even when its presence is previously known.

While not losing sight of the seriousness of past outbreaks, we believe that a great number of recent announcements and the resulting popular anxiety have been publicly carried many stages too far. With the coming years, improved social conditions and bettered health should rapidly turn the scale against the *B. Influenzæ* of Pfeiffer. In the approaching era we confidently expect a speedy ending of his reign of terror.

